# The Importance of Cultural Traits in Children’s Willingness to Provide Informal Care to a Parent

**DOI:** 10.1093/geroni/igaa057.1850

**Published:** 2020-12-16

**Authors:** Freya Diederich, Hans-Helmut König, Christian Brettschneider

**Affiliations:** 1 University Medical Center Hamburg-Eppendorf, Hamburg Center for Health Economics, Hamburg, Germany; 2 University Medical Center Hamburg-Eppendorf, Hamburg Center for Health Economics, Hamburg, Hamburg, Germany; 3 University Medical Center Hamburg-Eppendorf, Hamburg, Hamburg, Germany

## Abstract

The likelihood that a child will provide informal care to a parent varies across countries and between social groups within countries. We highlight the importance of cultural traits in children’s value of informal care and their willingness to provide informal care to a parent. We initially construct a cultural measure of the strength of family ties at the country level using data from the World Values Survey. Then, we use a sample of second-generation immigrants from the German Family Panel (N=1,041) and regress their value of informal care on the strength of family ties that prevails in their parents’ country of origin. Immigrants who have origins in countries with strong family ties are significantly more likely to report a high value of informal care. Finally, we show that children who report a high value of informal care are significantly more likely to provide informal care to a parent in need. Part of a symposium sponsored by the International Aging and Migration Interest Group.

